# Follistatin-like protein 1 is elevated in systemic autoimmune diseases and correlated with disease activity in patients with rheumatoid arthritis

**DOI:** 10.1186/ar3241

**Published:** 2011-02-08

**Authors:** Dawei Li, Yuji Wang, Nanwei Xu, Qianghua Wei, Min Wu, Xiaofeng Li, Ping Zheng, Sai Sun, Yuli Jin, Gailian Zhang, Ruomin Liao, Ping Zhang

**Affiliations:** 1 State Key Laboratory of Genetic Engineering, Institute of Genetics, School of Life Sciences, Fudan University, 220 Handan Road, Shanghai, 200433, People's Republic of China; 2 Department of Orthopaedics, the Affiliated Hospital of Nanjing Medical University, Changzhou second People's Hospital, 29 Xinglong Alley, Changzhou, 213003, People's Republic of China; 3 Department of Rheumatology, Shanghai First People's Hospital Affiliated to Shanghai Jiaotong University, 85 Wujin Road, Shanghai, 200080, People's Republic of China; 4 Department of Rheumatology and Immunology, The Third Affiliated Hospital, Suzhou University, Changzhou first People's Hospital, 185 Juqian Street, Changzhou, 213003, People's Republic of China; 5 Department of Rheumatology, the Second Hospital, Shanxi Medical University, 382 Wuyi Road, Taiyuan, 030001, People's Republic of China; 6 Department of Gastroenterology, Shanghai First People's Hospital Affiliated to Shanghai Jiaotong University, 85 Wujin Road, Shanghai, 200080, People's Republic of China; 7 Department of Respiratory Medicine, Shanghai First People's Hospital Affiliated to Shanghai Jiaotong University, 85 Wujin Road, Shanghai, 200080, People's Republic of China; 8 Clinical laboratory, the Affiliated Hospital of Nanjing Medical University, Changzhou Second People's Hospital, 29 Xinglong Alley, Changzhou, 213003, People's Republic of China

## Abstract

**Introduction:**

Follistatin-like protein 1 (FSTL1) is a proinflammation mediator implicated in arthritis in rodent animal models. The present study is aimed at assessing FSTL1 levels in systemic autoimmune diseases and correlating them with disease activity in patients with rheumatoid arthritis (RA).

**Methods:**

Serum FSTL1 levels from 487 patients with systemic autoimmune diseases and 69 healthy individuals were measured by enzyme-linked immunosorbent assay (ELISA). FSTL1 expression in synovial fluid (SF) and synovial tissues (STs) was determined by ELISA, immunohistochemistry, real-time polymerase chain reaction (RT-PCR) and western blot analysis in RA patients and trauma controls. FSTL1 levels in fibroblast-like synoviocytes (FLSs) from RA patients were determined by real-time PCR and western blot analysis.

**Results:**

Serum FSTL1 levels were significantly elevated in patients with RA, ulcerative colitis, systemic lupus erythematosus, Sjögren's syndrome (SS), systemic sclerosis and polymyositis/dermatomyositis. Serum FSTL1 levels in the RA and secondary SS patients were substantially higher than those in other patients. Serum FSTL1 levels were increased in early RA, rheumatoid factor (RF)- and anti-cyclic citrullinated peptide antibody (ACPA)-negative patients compared to healthy controls. Moreover, serum FSTL1 concentrations were significantly higher in long-standing RA patients than in early RA patients and in the RF- and ACPA-positive RA patients than in RF- and ACPA-negative RA patients. Elevated FSTL1 levels in the STs and SF of RA patients were also observed. FSTL1 levels in serum were markedly higher than those in SF in RA patients. The strongest FSTL1 staining was detected in the cytoplasm of synovial and capillary endothelial cells from RA synovium. Furthermore, FSTL1 was induced in FLSs by inflammatory mediators. Importantly, serum FSTL1 levels were correlated with several important biologic and clinical markers of disease activity, including erythrocyte sedimentation rate, C-reactive protein, RF, ACPA, swollen joint count, patient global visual analogue scale score and Disease Activity Score 28 in the adult RA patient population. Notably, serum FSTL1 levels were significantly diminished following successful treatment and clinical improvement.

**Conclusions:**

Elevated FSTL1 levels reflect not only joint diseases but also inflammation and tissue degradation in systemic autoimmune diseases. Serum FSTL1 levels may thus serve as a serological inflammatory marker of disease activity in RA patients.

## Introduction

Follistatin-like protein 1 (FSTL1) is a secreted glycoprotein with extensive glycosylation modifications and exists in two isoforms that differ in the extent of sialylation [1]. It is widely expressed in all organs [2] and is also detectable in the medium of cardiac myocytes [3] and endothelial cells (ECs) [4]. FSTL1 expression is upregulated in cardiac and skeleton myocytes in response to ischemic stress [4] and in the osteoblast cell line stimulated with proinflammation cytokines [5]. It has been shown that FSTL1 functions as an antiapoptotic protein by increasing both Akt and extracellular signal-regulated kinase activities [3]. FSTL1 promotes EC function and stimulates revascularization through activation of the Akt-endothelial nitric oxide synthase signaling pathway [4]. FSTL1 serum concentrations have been assessed in healthy individuals and in patients with acute coronary syndrome and were found to correlate with disease mortality during follow-up [6].

Rheumatoid arthritis (RA) is characterized by persistent multiple synovial inflammation and joint destruction. FSTL1 has also been reported to be involved in the pathogenesis of RA. Tanaka *et al. *[7] first identified it as an autoantigen when FSTL1 autoantibodies were found in the serum and synovial fluid (SF) of RA patients. Furthermore, FSTL1 mRNA is upregulated in the RA synovium [8] and the inflammatory synovial pannus of the collagen-induced arthritis (CIA) mouse [9]. Recently, it has been demonstrated that FSTL1 is a novel proinflammatory molecule. Overexpression of FSTL1 in macrophages and fibroblasts augments the activity of proinflammatory cytokines, including interleukin (IL)-1β, tumor necrosis factor α (TNFα), and IL-6 and causes severe arthritis in the normal mouse [10]. FSTL1 neutralization was shown to ameliorate arthritis by inhibiting production of interferon (IFN)-γ and chemokine (C-X-C motif) ligand 10 in arthritic joints of CIA mice [5]. The aims of the present study were to determine FSTL1 levels in patients with systemic autoimmune diseases and to further assess the relationship between serum FSTL1 levels and RA disease progression.

## Materials and methods

### Subjects

Peripheral blood was collected by venipuncture from patients with systemic autoimmune diseases, comprising the following: 207 RA, 22 reactive arthritis (ReA), 34 psoriatic arthritis (PsA), 33 ankylosing spondylitis (AS), 20 Crohn's disease (CD), 22 ulcerative colitis (UC), 40 systemic lupus erythematosus (SLE), 16 primary and 28 secondary Sjögren's syndrome (pSS and sSS, respectively), 22 systemic sclerosis (SSc) and 43 polymyositis/dermatomyositis (PM/DM). All patients included in this study were diagnosed according to the respective diagnostic criteria for those diseases [11-19]. We included a total of 69 apparently healthy individuals (HC) without regular medication or chronic drug or alcohol abuse or other chronic disease or acute illness. Their demographic, clinical and laboratory characteristics are summarized in Tables 1 and 2. Serum was acquired and stored immediately at -20°C. Erythrocyte sedimentation rate (ESR) was measured by the Westergren method. Serum C-reactive protein (CRP) and rheumatoid factor (RF) levels were determined in the clinical laboratory using a latex photometric immunoassay. Anti-cyclic citrullinated peptide (CCP) antibodies (ACPAs) were detected using a commercial anti-CCP2 enzyme-linked immunosorbent assay (ELISA) kit (Fuchun Kexin Biotech, Shanghai, China) following the manufacturer's instructions. Among 207 RA patients, RF-positive patients (RF >20 IU/ml) and ACPA-positive patients (ACPA >50 RU/ml) accounted for 71.5% and 73.8% of the RA population, respectively. There were 48 early RA patients (disease duration ≤6 months) who accounted for 23.2% of the RA population. In addition, 81 patients were assessed by a composite Disease Activity Score 28 (DAS28) [20] using the ESR, the numbers of swollen and tender joints and the global assessment of the patients' disease activity. We recruited RA patients without treatment or treated with disease-modifying antirheumatic drugs (DMARDs), biological drugs and corticosteroids alone or in combination. Finally, 20 of these patients were successfully followed up after 6 months of treatment with infliximab and/or DMARDs. The efficacy assessment for each patient was based on the ACR preliminary definition of improvement in RA. Responders achieved at least an ACR20 response (that is, improvement of ≥20% according to the American College of Rheumatology (ACR) response criteria). Nonresponders did not achieve an ACR20 response.

**Table 1:** Characteristics and serum FSTL1 levels of the subjects investigated^a^

Characteristics	RA	ReA	PsA	AS	CD	UC	SLE	pSS	sSS	SSc	PM/DM	HC
Total number of subjects	207	22	34	33	20	22	40	16	28	22	43	69
Age^b^, years	54	23.5	46.5	44	33	35	37	50	53	53.5	41	38
25th percentile	43.5	13.8	33	29.5	25.5	29.3	31.3	48	39.3	43.5	26	27
75th percentile	63	33.5	54	51	43	45.8	52.3	63	63	58	58	41
Number of female/male subjects	159/48	8/14	15/19	9/24	6/14	9/13	34/6	14/2	24/4	17/5	31/12	37/32
Disease duration^b^, years	3	20 days	5	2	3	4	4	2	3	2	1	-
25th percentile	0.5	13 days	3	1	1	2	1	1	1	1	0.3	-
75th percentile	10	75 days	7	4	6	7	6	4	8	5	2	-
Serum FSTL1 levels, μg/l												
Geometric mean	74.28	3.74	7.57	6.19	6.39	13.61	14.60	29.94	73.88	16.48	13.79	6.49
Lower 95% CI	60.89	2.73	4.99	5.03	5.35	9.71	10.49	15.76	45.79	7.81	9.97	5.89
Upper 95% CI	90.60	5.12	11.48	7.62	7.63	19.06	20.30	56.88	119.20	34.76	19.06	7.15
RSD (%)	129.17	104.58	134.90	63.33	39.06	112.35	115.27	148.22	117.81	133.00	186.28	36.71
Median	71.27	3.71	4.52	5.21	6.05	10.78	12.92	34.81	82.48	25.01	10.74	6.60
25th percentile	25.51	2.49	3.47	3.67	4.92	7.48	6.84	10.51	23.48	3.38	6.76	5.28
75th percentile	236.70	5.46	29.59	9.37	8.55	28.24	34.12	47.37	169.70	62.68	29.60	8.19
Above normal, %^c^	87.43	9.09	26.47	18.18	5	36.36	52.5	68.75	96.43	54.55	48.84	4.3
*P *versus HC	<0.001	<0.001	0.100	0.375	0.627	<0.001	<0.001	<0.001	<0.001	<0.001	0.018	-
*P *versus RA	-	<0.001	<0.001	<0.001	<0.001	<0.001	<0.001	0.013	0.937	<0.001	<0.001	<0.001

^a^FSTL1, follistatin-like protein 1; 95% CI, 95% confidence interval; -, not determined; RSD, relative standard deviation; HC, healthy individuals; RA, rheumatoid arthritis; ReA, reactive arthritis; PsA, psoriatic arthritis; AS, ankylosing spondylitis; CD, Crohn's disease; UC, ulcerative colitis; SLE, systemic lupus erythematosus; pSS, primary Sjögren's syndrome; sSS, secondary Sjögren's syndrome; SSc, systemic sclerosis; PM/DM, polymyositis/dermatomyositis; ^b^median; ^c^normal range denoted as mean + 2 SD was 12 μg/l.

**Table 2:** Disease characteristics of RA patients investigated^a^

Characteristics	*n*	Median (25th to 75th percentile)
ESR, mm/h	199	33 (17 to 61)
CRP, mg/l	207	8.1 (2.5 to 33.6)
RF, IU/ml	207	63.6 (15 to 185.5)
ACPA, RU/ml	206	251.9 (42.65 to 650.7)
Tender joint count	81	4 (2 to 7)
Swollen joint count	81	2 (1 to 5)
Patient global VAS score	81	50 (30 to 70)
DAS28 score	81	4.7 (3.4 to 5.6)

^a^RA, rheumatoid arthritis; ESR, erythrocyte sedimentation rate; CRP, C-reactive protein; RF, rheumatoid factor; IU, international units; RU, relative units; ACPA, anti-cyclic citrullinated peptide antibody; VAS, visual analogue scale; DAS28, disease activity score 28.

Synovial tissues (STs) from another cohort of 14 RA patients (median age, 53 years; median disease duration, 6.5 years; median DAS28 score, 4.3) were obtained by total knee joint replacement or arthroscopic synovectomy. Each sample was inspected visually to ensure that only inflamed tissue was included. Control samples were taken from 13 trauma patients with normal synovium. Most of tissue samples were split into three parts: one was stored at -70°C for mRNA and protein extraction, one was fixed in 4% paraformaldehyde and embedded in paraffin and one was minced and used for separation of synovial cells immediately upon acquisition. SF was collected from most of these patients during knee joint surgery.

Collection of ST, SF and peripheral blood samples was performed according to the medical ethics regulations of Nanjing Medical University. This study was approved by the Nanjing Medical University Review Board, and written permission was requested and received from all patients and healthy individuals in the study.

### Enzyme-linked immunosorbent assay

Serum FSTL1 levels were measured using a standard quantitative sandwich ELISA (Groundwork Biotechnology Diagnosticate, San Diego, CA, USA) with a 100 pg/ml detection limit of sensitivity. Serum and SF samples were diluted 1:5 and 1:10 in phosphate-buffered saline, respectively. Concentrations were reported as micrograms per liter. All analyses and calibrations were performed in duplicate. Optical densities were determined using an absorbance microplate reader (Elx808™ BioTek Instruments, Winooski, VT, USA) at 450 nm. Gen5 Data Analysis software (BioTek Instruments) was used to analyze all materials and depict the standard curve.

### Real-time PCR

Total RNA was extracted from minced cryogenic tissues or cultured fibroblast-like synoviocytes (FLSs) using the miRNeasy Mini Kit (Qiagen, Basel, Switzerland) according to the manufacturer's instructions. On-column digestion of contaminated DNA was performed using the RNase-Free DNase Set (Qiagen). Total RNA (2 μg) was reverse-transcribed using the ReverTra Ace kit (Toyobo, Osaka, Japan) according to the manufacturer's instructions. A quantitative real-time polymerase chain reaction (RT-PCR) assay was performed using SYBR Green (Toyobo) in an Applied Biosystems 7900HT Sequence Detection System (Applied Biosystems, Foster City, CA, USA). Primer sequences for amplifying FSTL1 cDNA and internal control glyceraldehyde 3-phosphate dehydrogenase (GAPDH) were as follows: FSTL1, 5'-CGATGGACACTGCAAAGAGA-3' (forward) and 5'-CCAGCCATCTGGAATGATCT-3' (reverse); GAPDH, 5'-AGGGCTGCTTTTAACTCTGGT-3' (forward) and 5'-CCCCACTTGATTTTGGAGGGA-3' (reverse). The comparative threshold cycle method was used for relative quantification of mRNA.

### Western blot analysis

Minced cryogenic tissues or cultured FLSs were lysed and boiled in sodium dodecyl sulfate (SDS) Lamini buffer. SDS-polyacrylamide gel electrophoresis was performed on 10% polyacrylamide gel and transferred to nitrocellulose membrane. Goat anti-human FSTL1 polyclonal antibody (R&D Systems, Minneapolis, MN, USA) and actin AC-40 (Sigma-Aldrich, St. Louis, MO, USA) were used to detect FSTL1 and β-actin expression, respectively.

### Immunohistochemistry

Immunohistochemical staining with polyclonal anti-FSTL1 antibody was performed on archival formalin-fixed, paraffin-embedded ST using the peroxidase technique. Briefly, sections were deparaffinized and rehydrated. Epitope retrieval was performed in citrate buffer (pH 6) in a water bath at 98°C for 35 minutes with cooling for 20 minutes before immunostaining. Tissues were incubated with the primary anti-FSTL1 antibody (1:400 dilution) overnight at 4°C after blocking and then exposed to biotinylated secondary linking antibody (Boster, Wuhan, China) for 20 minutes. Biotin detection was performed with peroxidase-conjugated streptavidin. Finally, the slides were incubated with 3,3'-diaminobenzidine (Boster) for about 5 to 10 minutes and with hematoxylin as a counterstain for 1 minute. Sections were washed between incubations with Tris-buffered saline buffer (pH 7.6). Goat polyclonal antibody immunoglobulin G (IgG) was used as a negative control throughout. Each section was examined independently by two investigators (DL and YW) in a blinded manner.

### Isolation and culture of human synovial fibroblasts

ST specimens were obtained from patients with RA or trauma by synovectomy or joint replacement surgery. These joint tissues were minced and incubated with 1 mg/ml collagenase I (Sigma-Aldrich, St. Louis, MO, USA) in serum-free Dulbecco's modified Eagle's medium (DMEM) (Gibco BRL, Grand Island, NY, USA) for 4 hours at 37°C, filtered through a nylon mesh, extensively washed and cultured in DMEM supplemented with 10% fetal calf serum (Gibco), 100 U of penicillin, 100 μg/ml streptomycin, and 2 mM L-glutamine in a humidified atmosphere containing 5% CO_2_. After overnight culture, the nonadherent cells were removed. The adherent cells were split at a ratio of 1:3 after these cells grew to 80% confluence. Synoviocytes were used from passages 4 through 6 in these experiments, during which they consisted of a homogeneous population of FLSs.

### Reagents and stimulation assays

Cultured FLSs were grown in 100-mm cell culture dishes (6 to 8 × 10^5 ^cells/dish). Cultures were stimulated for 24 hours for mRNA extraction or for 36 hours for protein collection with the following agents: 20 μg/ml polyinosinic:polycytidylic acid (InvivoGen, San Diego, CA, USA), 100 ng/ml lipopolysaccharide from *Escherichia coli *(Sigma-Aldrich), 300 ng/ml bacterial lipoprotein (InvivoGen), 10 ng/ml recombinant interleukin-1β (IL-1β) (Invitrogen, Carlsbad, CA, USA), 10 ng/ml tumor necrosis factor α (TNFα) (Invitrogen) and 10 ng/ml transforming factor β (TGFβ) (Invitrogen).

### Statistical analysis

Statistical analyses were performed using Prism and Instat software (GraphPad Software, San Diego, CA, USA) and SPSS 13.0 software (SPSS Inc., Chicago, IL, USA). The significance of the differences was evaluated using the Kruskal-Wallis test among multiple groups and the Mann-Whitney *U *test between groups. Follow-up data were evaluated by the Wilcoxon matched pairs test. The relationships between serum FSTL1 levels and other parameters were evaluated using Spearman's rank-correlation test. All quoted *P *values were two-tailed, and *P *values less than 0.05 were considered significant.

Serum FSTL1 levels that were not normally distributed were transformed to their natural logarithm (ln) for the regression analyses. Linear multiple regression analyses were performed with ln(FSTL1) levels as dependent variables and the raw data of CRP, ESR, RF, ACPA, disease duration, sex and age as independent variables. The test for significance of regression is carried out using the analysis of variance. The *t*-test was used to check the significance of individual regression coefficients in the multiple linear regression model. The level of significance was set at 0.05.

## Results

### Serum FSTL1 levels in patients with systemic autoimmune diseases

**Figure 1:**
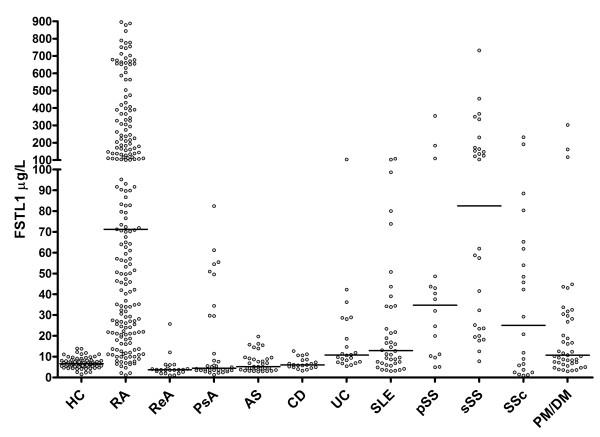
**Serum concentrations of FSTL1 in HC (*n *= 69) and patients with RA (*n *= 207), ReA (*n *= 22), PsA (*n *= 34), AS (*n *= 33), CD (*n *= 20), UC (*n *= 22), SLE (*n *= 40), pSS (*n *= 16), sSS (*n *= 28), SSc (*n *= 22) and PM/DM (*n *= 43)**. Horizontal lines represent median values. Each point represents an individual value. FSTL1, follistatin-like protein 1; HC, healthy individuals; RA, rheumatoid arthritis; ReA, reactive arthritis; PsA, psoriatic arthritis; AS, ankylosing spondylitis; CD, Crohn's disease; UC, ulcerative colitis; SLE, systemic lupus erythematosus; pSS, primary Sjögren's syndrome; sSS, secondary Sjögren's syndrome; SSc, systemic sclerosis; PM/DM, polymyositis/dermatomyositis.

Serum FSTL1 levels and their distributions were determined for the various groups and found to differ significantly between patient groups and HC (Figure 1 and Table 1; Figure S1 and Table S1 in Additional file 1). Serum FSTL1 levels in the RA and sSS groups were substantially higher than those found in the other patient groups and HC. Serum FSTL1 levels in the pSS, SLE, SSc, PM/DM and UC patient groups were also significantly higher than those in HC. No significant difference was observed in serum FSTL1 levels between the PsA, CD and AS patient groups and HC. However, serum FSTL1 concentrations in ReA were significantly lower than those in HC. UC patients showed higher serum FSTL1 levels than CD patients.

### Serum FSTL1 levels in the subgroups of RA patients

**Figure 2:**
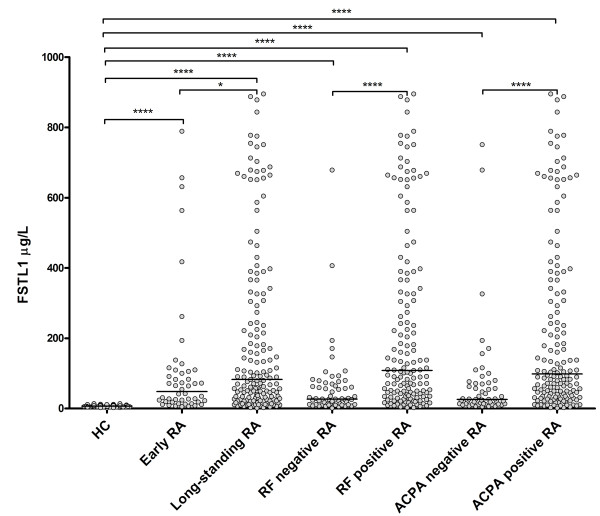
**Serum FSTL1 levels in HC (*n *= 69), early RA patients (*n *= 48), long-standing RA patients (*n *= 159), RF-negative RA patients (*n *= 59), RF-positive RA patients (*n *= 148), ACPA-negative patients (*n *= 54) and ACPA-positive patients (*n *= 152)**. Horizontal lines represent median values. Each point represents an individual value (*P < 0.05; ****P < 0.0001). FSTL1, follistatin-like protein 1; HC, healthy individuals; RA, rheumatoid arthritis; RF, rheumatoid factor; ACPA, anti-cyclic citrullinated peptide antibody.

Furthermore, we compared serum FSTL1 levels between early and long-standing RA patients (Figure 2). Early RA patients showed increased serum FSTL1 levels (median, 48.4 μg/l; 25th to 75th percentile, 18.6 to 108.4 μg/l) compared with HC. Serum FSTL1 levels in long-standing RA patients (median, 82.7 μg/l; 25th to 75th percentile, 28.2 to 307.5 μg/l) were higher than those in early RA patients and HC. We also compared serum FSTL1 levels of RF- and ACPA-positive RA patients with those of RF- and ACPA-negative RA patients (Figure 2). The serum FSTL1 levels in RF-negative RA patients (median, 27.1 μg/l, 25th to 75th percentile, 11.8 to 70.0 μg/l) and ACPA-negative RA patients (median, 25.8 μg/l, 25th to 75th percentile, 13.3 to 71.8 μg/l) were higher than those in HC. In addition, FSTL1 levels in the RF-positive patients (median, 108.8 μg/l, 25th to 75th percentile, 40.5 to 366.4 μg/l) and ACPA-positive patients (median, 98.3 μg/l, 25th to 75th percentile, 35.6 to 359.9 μg/l) were higher than those of their RF-negative and ACPA-negative counterparts.

### Elevated FSTL1 expression in ST and SF in patients with RA

**Figure 3:**
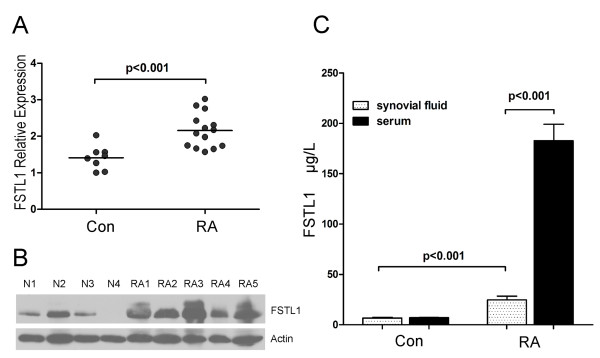
**(A) FSTL1 mRNA expression in cryogenic tissue samples from patients with RA (*n *= 14) and trauma controls (Con, *n *= 8)**. Results are expressed in relative units, and horizontal lines represent means. The *P *values derived using Student's *t*-test are indicated. **(B) **FSTL1 protein expression in representative tissue samples from trauma controls (N1-N4: N represents normal synovial tissues; 1-4 represents four different trauma control individuals) and RA patients (RA1-RA5, RA represents RA synovial tissues; 1-5 represents five different RA patients). FSTL1 immunoreactive band appears as a band with electrophoretic mobilities corresponding to 46 kDa. β-Actin was used as loading control. **(C) **FSTL1 concentrations in SF from trauma controls (*n *= 7) and RA patients (*n *= 13) and in serum from healthy individuals (*n *= 69) and RA patients (*n *= 207). Values are means ± SEM. FSTL1, follistatin-like protein 1; RA, rheumatoid arthritis; SF, synovial fluid.

It was previously reported that FSTL1 mRNA was overexpressed in STs of RA [8]. We confirmed these results and found that FSTL1 mRNA levels were increased by approximately 1.52-fold in STs from RA patients compared to the trauma controls (Figure 3A). Furthermore, the FSTL1 protein levels were analyzed from representative samples by Western blot analysis. Consistent with the elevation of its transcript, FSTL1 protein expression was markedly increased in RA patients' STs compared with those in the trauma controls (Figure 3B). The specificity of FSTL1 antibodies was confirmed because no nonspecific band was detected on immunoblot imaging studies. Furthermore, FSTL1 levels in SF from RA patients (mean ± SEM, 24.8 ± 3.6 μg/l) were significantly higher than those of the trauma controls (mean ± SEM, 6.59 ± 0.9 μg/l). Interestingly, serum FSTL1 levels (mean ± SEM, 182.8 ± 16.4 μg/l) were dramatically higher than SF FSTL1 levels in RA patients. In contrast, no difference was found between serum and SF FSTL1 levels in the control group (Figure 3C).

### Immunohistochemical localization of FSTL1 in STs in patients with RA

**Figure 4:**
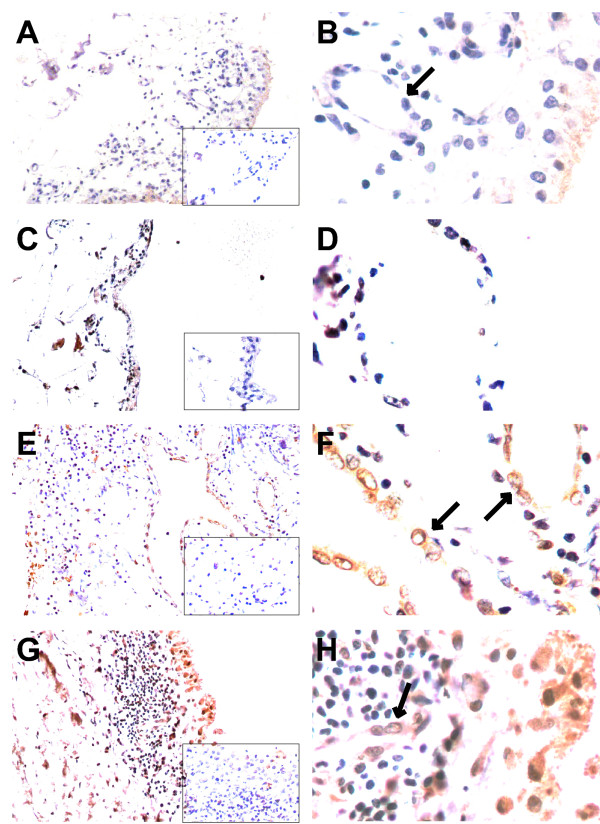
**Immunohistochemical detection of FSTL1 in synovial tissues**. **(A-D) **Trauma control synovial tissues with normal synovium. Synovial cells with little or weak FSTL1 immunostaining are shown. **(E-H) **RA synovial tissues. Hyperplastic synovial lining and sublining cells with positive FSTL1 immunostaining are shown. All tissues were counterstained with hematoxylin. **(A**, **C**, **E **and **G) **Original magnification, ×100. **(B**, **D**, **F **and **H) **Original magnification, ×400. The negative controls were stained with polyclonal goat IgG (original magnification, ×100) and are shown in the insets **(A-D)**. Arrows indicate capillary endothelial cells from trauma control with normal synovial tissues **(B) **and RA synovial tissues **(F **and **H)**. FSTL1, follistatin-like protein 1; RA, rheumatoid arthritis; IgG, immunoglobulin G.

We next assessed FSTL1 tissue distributions in STs from RA patients and trauma controls (Figure 4). In normal synovium from trauma controls, FSTL1 staining was weak but positive in comparison to the IgG control, suggesting that FSTL1 was constitutively expressed, albeit at relatively low levels, in these tissues. A significant increase in FSTL1 staining was seen in STs from RA patients compared with trauma controls. FSTL1 staining was mainly seen in the cytoplasm of synoviocytes from lining and sublining, the major cell types present in synovium. FSTL1 expression was also evident in ECs of capillaries from STs of RA patients, but was much weaker and even absent in ECs from normal STs of trauma controls. In contrast, FSTL1 was not detectable in most infiltrated lymphocytes.

### Increased FSTL1 expression in FLSs from RA patients induced by proinflammatory mediators

**Figure 5:**
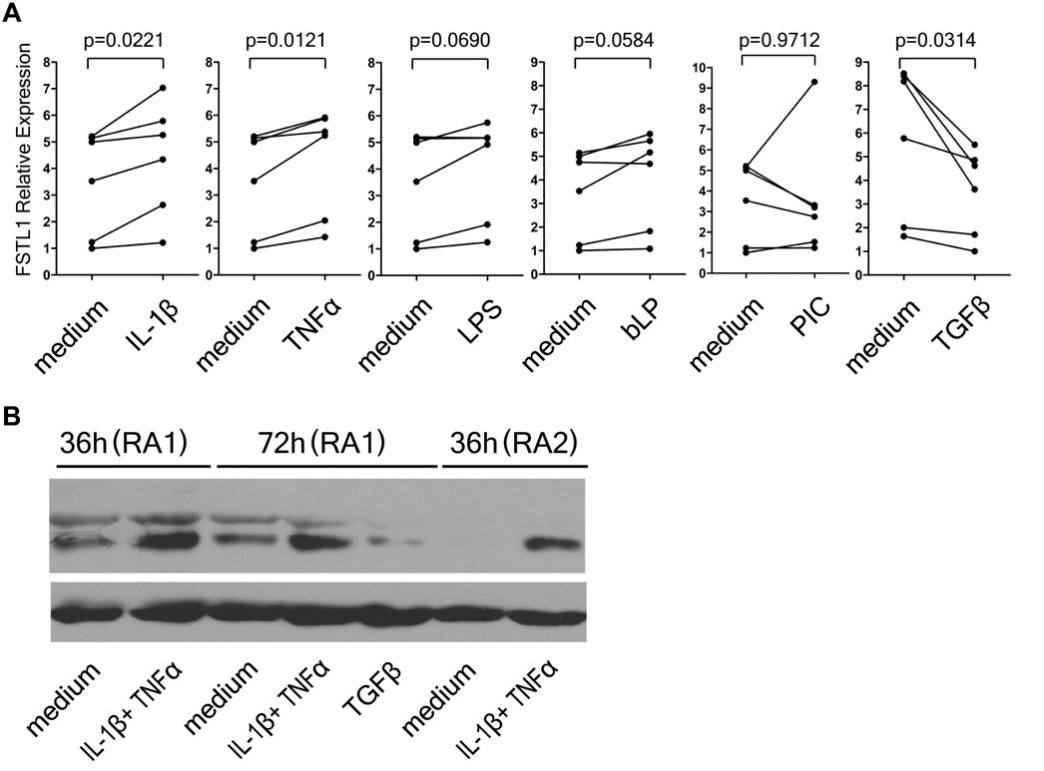
**Upregulation of FSTL1 expression induced by inflammatory mediators in fibroblast-like synoviocytes (FLSs) from patients with RA**. **(A) **RA FLSs (*n *= 6) were treated with the indicated stimuli for 24 hours or were left untreated (medium). FSTL1 mRNA was determined using RT-PCR. The results are presented in relative units, and the *P *values derived from paired *t*-tests are indicated for each comparison. **(B) **FLSs from two RA patients (RA1 and RA2) were stimulated by combined IL-1β and TNFα or by TGFβ, or they were left untreated for 36 hours or 72 hours. Cell lysates were collected and analyzed by Western blot analysis using the anti-FSTL1 polyclonal antibody. β-Actin was used as a loading control. FSTL1, follistatin-like protein 1; RA, rheumatoid arthritis; PCR, polymerase chain reaction; IL-1β, interleukin-1β; TNFα, tumor necrosis factor α; TGFβ, transforming growth factor β; LPS, lipopolysaccharide; bLP, bacterial lipoprotein; PIC, polyinosinic:polycytidylic acid.

To determine whether FLSs are a source of tissue for FSTL1, we separated primary FLSs and compared FSTL1 expression in FLSs from RA patients and trauma controls. No difference in FSTL1 levels was seen in FLSs (data not shown), suggesting that FSTL1 production in FLSs is dependent on an inflammatory milieu. Next, we selected six primary FLSs derived from different RA patients and stimulated them with inflammatory mediators. The FSTL1 transcript was significantly induced by IL-1β and TNFα in primary FLSs and was inhibited by TGFβ (Figure 5A). Consistently, FSTL1 protein expression was increased by combined IL-1β and TNFα treatment and inhibited by TGFβ in primary RA FLSs (Figure 5B).

### Serum FSTL1 levels correlated with parameters of disease activity in patients with RA

**Figure 6:**
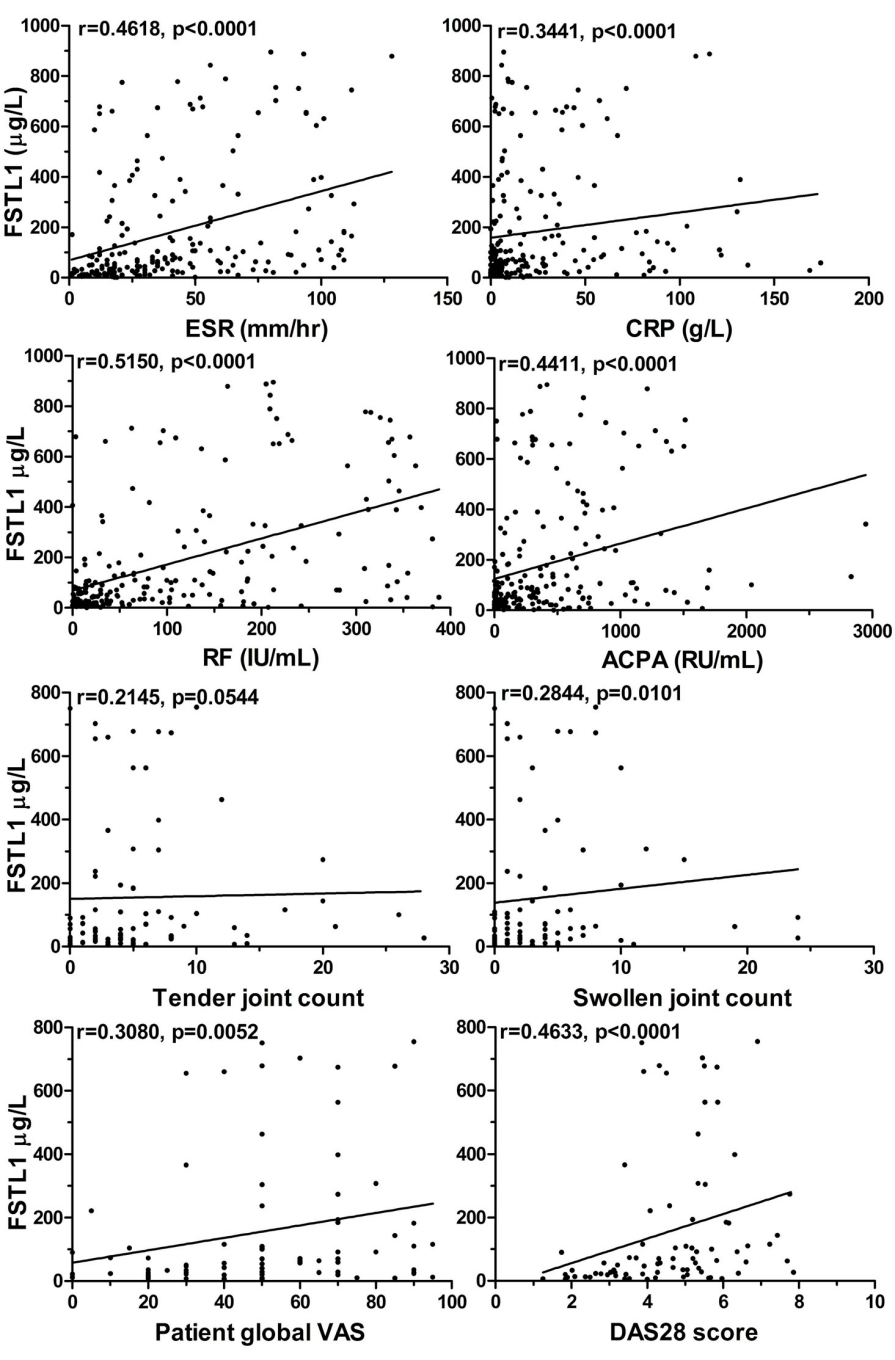
**Correlation of serum FSTL1 levels with ESR (*n *= 199), CRP (*n *= 207), RF (*n *= 207), ACPA (*n *= 206), tender (*n *= 81) and swollen (*n *= 81) joint counts, patient global VAS score (*n *= 81) and patient DAS28 score (*n *= 81) in adult RA population**. Correlation coefficients (*r*) and *P *values from the Spearman rank-order test are displayed. Each point represents an individual value. FSTL1, follistatin-like protein 1; ESR, erythrocyte sedimentation rate; CRP, C-reactive protein; RF, rheumatoid factor; ACPA, anti-cyclic citrullinated peptide antibody; VAS, visual analogue scale; DAS28, Disease Activity Score 28; RA, rheumatoid arthritis.

A significant positive correlation was seen between serum FSTL1 levels and serologic parameters of disease activity, including ESR, CRP, RF and ACPA, with levels of clinical parameters including swollen joint count and patient global VAS and, importantly, with disease activity, as indicated by the DAS28 score in the adult RA population (Figure 6). In a multiple linear regression analysis that used ln(FSTL1) as the dependent variable, older age, female sex, ESR, RF and ACPA were independently associated with FSTL1 (Table 3). The total *r*^2 ^value of this model was 0.45.

**Table 3:** Independent association of FSTL1 with baseline characteristics in 198 RA patients^a^

Characteristics	B	*P *value
Age, years	0.022	0.000
Sex	-0.594	0.003
Disease duration, years	-0.001	0.945
ESR, mm/h	0.012	0.001
CRP, mg/l	-0.001	0.649
RF, IU/ml	0.005	0.000
ACPA, RU/ml	0.001	0.002

^a^Multiple linear regression analysis association with the natural logarithm of follistatin-like protein 1 (FSTL1); RA, rheumatoid arthritis; B, unstandardized B coefficient; ESR, erythrocyte sedimentation rate; CRP, C-reactive protein; RF, rheumatoid factor; IU, international units; RU, relative units; ACPA, anti-cyclic citrullinated peptide antibody.

### Effects of treatment on serum FSTL1 levels in patients with RA

**Figure 7:**
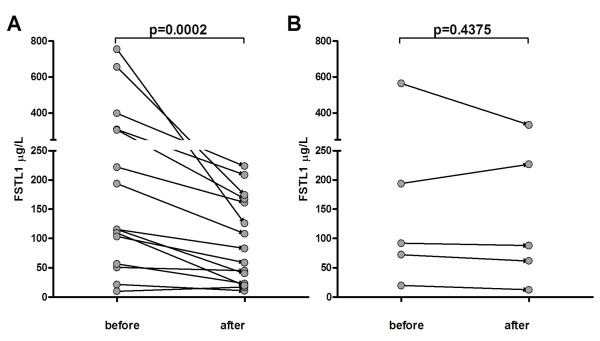
**Serum levels of FSTL1 in RA patients before and after treatment**. Paired serum samples were obtained from the 20 patients before treatment and after 6 months of treatment with DMARDs and/or infliximab. **(A) **Fifteen patients achieved at least an ACR20 response. **(B) **Five nonresponders did not achieve an ACR20 response. Each point represents an individual value. FSTL1, follistatin-like protein 1; RA, rheumatoid arthritis; DMARDs, disease-modifying antirheumatic drugs; ACR20, improvement of ≥20% according to the American College of Rheumatology (ACR) response criteria.

We performed follow-up studies of serum FSTL1 levels in RA patients after treatment with infliximab and/or DMARDs for 6 months (Figure 7). Among the 20 RA patients enrolled in the follow-up studies were four with ACR20, seven with ACR50, four with ACR70, and five nonresponders. Serum FSTL1 levels in 14 patients with active RA were significantly diminished following clinical improvement with treatment (infliximab alone in two patients, infliximab plus leflunomide in four patients, infliximab plus leflunomide plus methotrexate in two patients and leflunomide alone in six patients). Only one early stage patient treated with leflunomide alone who achieved an ACR20 response had a slight increase in serum FSTL1 concentrations (10.0 μg/l before treatment and 17.0 μg/l after treatment). Importantly, for the five patients who did not achieve an ACR20 response, serum FSTL1 levels did not change significantly before and after treatment.

## Discussion

In this study, we have found for the first time that serum FSTL1 levels are significantly elevated in patients with systemic autoimmune diseases, including RA, UC, SLE, SS, SSc and PM/DM. Furthermore, we have revealed that serum FSTL1 levels are correlated with serologic and clinical features of disease activity in the RA patient population. Finally, we have observed that serum FSTL1 levels are significantly decreased in RA patients following successful treatment and clinical improvement. These observations suggest that FSTL1 can serve as a reliable serological marker for disease activity in the RA patient population.

We also systemically assessed FSTL1 expression in adult RA patients for the first time. We found that FSTL1 levels were also increased in the STs and SF of RA patients. The strongest FSTL1 staining was detected in the cytoplasm of synovial and capillary ECs from RA synovium. Interestingly, we found that FSTL1 concentrations in serum were higher than those in synovial fluid in the adult RA population, suggesting that the source of FSTL1 was not limited to the synovium. In fact, previous reports have shown that FSTL1 is widely expressed in almost all organs, especially the lung and heart in rat [2]. Moreover, FSTL1 is also referred as a "myokine" because it is secreted into the peripheral blood by cardiac and skeletal muscle [3,4]. Oshima *et al. *[3] reported that FSTL1 is also secreted by ECs. Our results indicate that FSTL1 stained strongly in inflammatory ECs. These data suggest that ECs are a potential source of serum FSTL1, especially for those RA patients with complications such as rheumatoid vasculitis, pericarditis and pleuritis. However, we did observe that FSTL1 stained strongly in synovial cells and was induced in FLS by proinflammatory cytokines from RA patients. Therefore, we proposed that FLS is also the source of elevated FSTL1 levels, at least in STs. For these RA patients with lack of systemic arthritis symptoms, we could not exclude the possibility that FLS stimulated continually by proinflammatory mediators increased FSTL1 concentrations in STs and then released FSTL1 into serum.

A variety of autoantibodies exist, and microvascular EC injury frequently occurs among systemic autoimmune diseases, including RA, SLE, SS, SSc and PM/DM. It has been recognized that EC damage is caused by circulating immune complexes containing RF and other autoantibodies that form deposits in vessel walls, where they trigger an inflammatory reaction that leads to EC injury [21,22]. Previous reports have shown that FSTL1 was secreted by ECs [4]. In agreement with those observations, we found that FSTL1 stains strongly in inflammatory ECs. These findings raised the possibility that inflammatory ECs are responsible for high serum FSTL1 levels in those diseases. In addition, FSTL1 is also known to be secreted into the peripheral blood by cardiac and skeletal muscle, especially by ischemic muscle in a mouse model [3,4]. Skeletal muscle inflammation and injury could also lead to higher serum FSTL1 levels in PM/DM. However, serum FSTL1 levels were not elevated in seronegative spondyloarthropathy, including AS, PsA and ReA, possibly because of less frequent autoantibody-mediated vascular injury and vasculitis. It remains to be seen whether elevated serum FSTL1 levels found in systemic autoimmune diseases are relevant to the autoimmune response underlying the disease process.

Serum FSTL1 levels in RA and sSS patients were substantially higher than those in other patients. In fact, the disease of most sSS patients was secondary to RA in the present study. This finding may be explained by both persistent synovitis of multiple joints and frequent extraarticular manifestations in RA patients. Increased serum FSTL1 levels could reflect not only chronic multiple synovial joint inflammation but also systemic inflammation and tissue degradation.

Although the effects of increased FSTL1 on the inflammation process were not assessed in this study, it has been recently reported that FSTL1 is a novel proinflammatory mediator in a rodent animal model [5,10]. High circulating FSTL1 levels in RA patients might possibly cause deterioration in patients with arthritis by promoting production of proinflammatory cytokines and chemokines in arthritic joints. Another possibility is that FSTL1 promotes arthritis by increasing neovascularization of inflammatory synovium. It has been established that angiogenesis is associated with increased synovial inflammation and pannus formation and that blocking angiogenesis suppresses arthritis [23,24]. Previous studies have shown that FSTL1 overexpression enhances revascularization in mouse ischemic limbs [4]. Therefore, increased FSTL1 levels in RA could aggravate arthritis by FSTL1-mediated neovascularization in RA STs. However, some other reports have shown that FSTL1 plays immunosuppressive and immunomodulatory roles on immunity and inflammation. FSTL1 plays an immunomodulatory role in heart allograft transplantation [25] and ameliorates arthritis in mouse models [26,27]. More recently, Adams *et al. *[28] reported that FSTL1 protected the kidney from acute nephrotoxic injury by inhibiting IL-1β expression. Therefore, it could be that FSTL1 plays different roles in varied cell and disease settings or at different stages in the pathogenesis and progression of arthritis. It remains to be determined whether different serum and tissue FSTL1 levels play different roles in the adult RA population. However, our findings that elevated serum FSTL1 levels and their correlations with inflammatory status in patients with RA suggest its proinflammatory effects, at least in the adult RA population.

Our data also reveal that serum FSTL1 levels correlate with several important biologic and clinical markers of disease activity, including ESR, CRP, swollen joint count, patient global VAS and DAS28 score in the RA population. Importantly, serum FSTL1 levels decreased markedly following clinical improvement. These data indicate that serum FSTL1 could serve as a new biomarker for disease activity in RA patients. A recent report has shown that serum FSTL1 levels are slightly elevated in systemic onset juvenile rheumatoid arthritis. However, it is premature to conclude that FSTL1 represents a biomarker for the disease, because only small samples with little clinical information were assessed in that recent study [29].

In comparison to the existing markers, such as ESR and CRP, serum FSTL1 was produced, at least partially, at local inflammation sites, including arthritic joints and blood vessels, which could reflect the status of tissue damage more accurately than those traditional markers. In addition, it has been observed in the clinic that some patients have normal or low levels of ESR or CRP despite extensive arthritis [30]. Serum FSTL1 levels in these patients could be a useful inflammatory marker. Serum FSTL1 levels were independently related to titers of RF and ACPA, supporting the hypothesis that FSTL1 could be induced by immune complex-mediated vascular and tissue damage. In addition, RF and ACPA are correlated with a worse prognosis and the presence of aggressive disease [31]. Further study is required to assess the relationship between serum FSTL1 levels and joint damage and radiological progression in the RA patient population.

## Conclusions

FSTL1 levels are increased in systemic autoimmune diseases. Serum FSTL1 levels reflect the inflammatory status in RA patients. These results suggest that FSTL1 may serve as a novel biomarker for RA disease activity, raising the possibility that FSTL1 levels may be potential therapeutic targets in these diseases.

## Abbreviations

ACPA: anti-cyclic citrullinated peptide antibodies; AS: ankylosing spondylitis; bLP: bacterial lipoprotein; CD: Crohn's disease; DAS28: Disease Activity Score using 28 joint counts; DMARDs: disease-modifying antirheumatic drugs; EC: endothelial cell; ELISA: enzyme-linked immunosorbent assay; FLS: fibroblast-like synoviocyte; FSTL1: follistatin-like protein 1; HC: healthy control; IL-1β: interleukin-1β; LPS: lipopolysaccharide; PIC: polyinosinic:polycytidylic acid; PM/DM: polymyositis/dermatomyositis; PsA: psoriatic arthritis; RA: rheumatoid arthritis; ReA: reactive arthritis; SF: synovial fluid; SLE: systemic lupus erythematosus; SS: Sjögren's syndrome; SSc: systemic sclerosis; ST: synovial tissue; TGFβ: transforming factor β; TNFα: tumor necrosis factor α; UC: ulcerative colitis; VAS: visual analog scale.

## Competing interests

The authors declare that they have no competing interests.

## Authors' contributions

DL conceived and designed the study, interpreted and analyzed data and drafted and wrote the manuscript. DL and YW carried out the experiments. YW, DL, MW, XL, QW, P Zheng, SS, NX, YJ, GZ, RL and P Zhang completed the acquisition or preparation of clinical samples. All authors read and approved the final manuscript.

## Supplementary Material

Additional file 1 **Supplementary data**. Figure S1. Distribution of serum follistatin-like protein 1 (FSTL1) levels in the different groups including the healthy controls and the patients with systemic autoimmune diseases. Table S1. Descriptive statistics of serum follistatin-like protein 1 (FSTL1) levels in the different groups including the healthy controls and the patients with systemic autoimmune diseases. Click here for file
