# Supplementary material for: Follistatin-like protein 1 is elevated in systemic autoimmune diseases and correlated with disease activity in patients with rheumatoid arthritis
Source: Arthritis Res Ther. 2011 Feb 8;13(1):R17. doi: 10.1186/ar3241 (PMC3241361; doi:10.1186/ar3241)
Supplement: Additional file 1 — Supplementary data. Figure S1. Distribution of serum follistatin-like protein 1 (FSTL1) levels in the different groups including the healthy controls and the patients with systemic autoimmune diseases. Table S1. Descriptive statistics of serum follistatin-like protein 1 (FSTL1) levels in the different groups including the healthy controls and the patients with systemic autoimmune diseases. [file ar3241-S1.DOC]

**Supplementary materials**

**Supplementary Figure S1.**

**
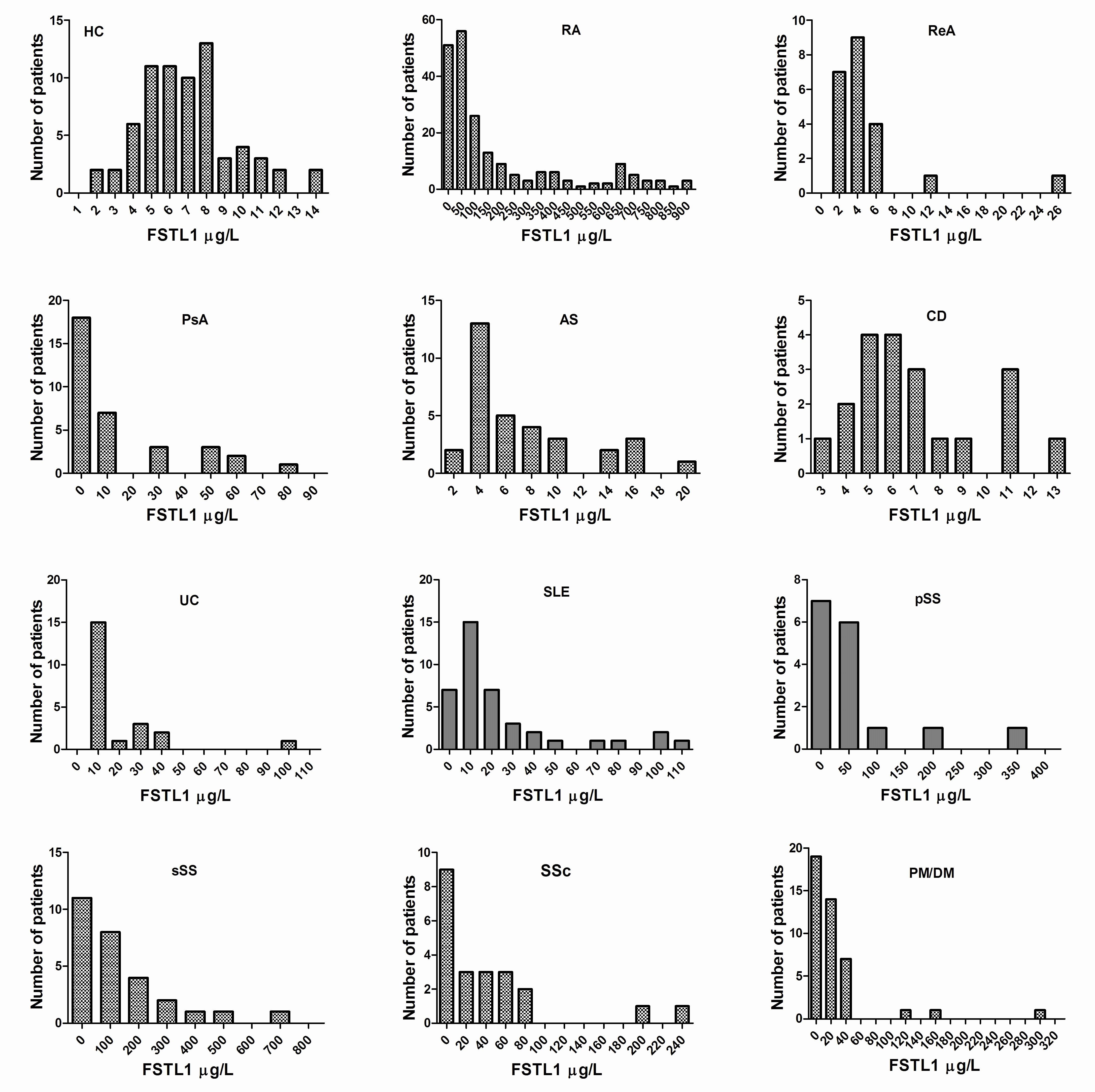
**

**Figure S1.** Distribution of serum FSTL1 concentrations in HC (n=69) and in patients with RA (n=207), ReA (n=22), PsA (n=34), AS (n=33), CD (n=20), UC (n=22), SLE (n=40), pSS (n=16), sSS (n=28), SSc (n=22) and PM/DM (n=43). HC: healthy individuals; RA: rheumatoid arthritis; ReA: reactive arthritis; PsA: psoriatic arthritis; AS: ankylosing spondylitis; CD: Crohn's disease; UC: ulcerative colitis; SLE: systemic lupus erythematosus; pSS: primary Sjogren's syndrome; sSS: secondary Sjogren's syndrome; SSc: systemic sclerosis; PM/DM: polymyositis/dermatomyositis.

**Supplementary Table S1**

**Table S1.** Descriptive statistics of serum FSTL1 levels in the different groups

|  | ReA | PsA | AS | CD | UC | SLE | pSS | sSS | SSc | PM/DM |
| --- | --- | --- | --- | --- | --- | --- | --- | --- | --- | --- |
|  | n=22 | n=34 | n=33 | n=20 | n=22 | n=40 | n=16 | n=28 | n=22 | n=43 |
| ReA |  | 0.0825 | 0.0068 | 0.0014 | < 0.0001 | <0.0001 | <0.0001 | <0.0001 | 0.0043 | <0.0001 |
| PsA |  |  | 0.7396 | 0.2479 | 0.0061 | 0.0049 | 0.0014 | <0.0001 | 0.0825 | 0.0071 |
| AS |  |  |  | 0.6074 | 0.0003 | 0.0003 | <0.0001 | <0.0001 | 0.0298 | 0.0003 |
| CD |  |  |  |  | 0.0002 | 0.0015 | 0.0001 | <0.0001 | 0.0606 | 0.0028 |
| UC |  |  |  |  |  | 0.8772 | 0.0276 | <0.0001 | 0.4887 | 0.8843 |
| SLE |  |  |  |  |  |  | 0.0362 | <0.0001 | 0.6643 | 0.6784 |
| pSS |  |  |  |  |  |  |  | 0.0429 | 0.5059 | 0.0166 |
| sSS |  |  |  |  |  |  |  |  | 0.0042 | <0.0001 |
| SSc |  |  |  |  |  |  |  |  |  | 0.4583 |

The *p* values from the Mann-Whitney U test between groups are displayed.
